# Bioinformatics approach to identify potential biomarker and drug target for the clinical and subclinical mastitis disease in dairy cattle

**DOI:** 10.1371/journal.pone.0349172

**Published:** 2026-05-13

**Authors:** Md. Rabiul Auwul

**Affiliations:** Department of Agricultural and Applied Statistics, Faculty of Agricultural Economics and Rural Development, Gazipur Agricultural University, Gazipur, Bangladesh; Sri Venkateswara Veterinary University, INDIA

## Abstract

Mastitis in dairy cattle is a serious issue that affects not just the animals but also has broad social, cultural, economic, and human consequences. It does in a wide variety of ways and the most remarkable of which are reduced milk yield and produce poor milk quality. This study takes an approach of bioinformatics to track down new targets and biomarkers which can be used to diagnose the clinical and subclinical forms of mastitis and at the same time find the way to treat and manage the disease. Comparing genes that express at a different level and the protein network, we identified three key genes (CDKN1A, FKBP5 and SLC7A5) and pathways that mastitis includes both in clinical and subclinical form. In functional term, multicellular organismal process regulation, cell population proliferation, protein binding are identified as critical biological processes. Additionally, machine learning algorithms applied to validate the identified candidate biomarkers. Potential repurposing drug targets are identified based on the commonly selected differentially expressed genes. This integrative approach not only provides insights into the molecular mechanisms underlying mastitis but also offers a robust framework for developing targeted therapies and diagnostic tools, ultimately contributing to better herd health and productivity. The findings from this study pave the way for precision veterinary medicine, with the ability to decrease the impact of the economic burden of mastitis on the dairy industry.

## 1. Introduction

Mastitis is one of worldwide and economically important diseases facing the dairy cattle industry. Primarily because bacteria infect the mammary glands leading resulting decrease in milk production as well as changes in its composition, thereby resulting into substantial economic losses to farmers growing them. Globally more than 80% of total world’s cattle milk comes from various countries [1]. Mastitis has become an endemic issue within global dairy sector as well as some cases assaulting cows, goats and buffaloes among others with mastitis affecting these breeds too. The illness can typically be grouped into two main types namely, clinical mastitis which shows visible signs of inflammation like swollen red painful udders containing abnormal looking fluids; subclinical mastitis where there aren’t any notable clinical signs but affects both quality and quantity accordingly. Several reasons lead to mastitis prevalence namely: poor management practices, limited veterinary services, significant environmental contamination and incorrect milking method as well lack of awareness amongst farmers.

Early identification and prompt intervention for mastitis are indispensable in minimizing its effect on the volume as well as quality of milk. The present diagnostic systems depend on somatic cell count (SCC) as well California Mastitis Test (CMT), which serves to measure inflammation indirectly from milk specimens [2,3]. The traditional method for diagnosis and treatment of mastitis included standard bacteriological techniques, which take time and often do not recognize the aetiological agents in subclinical cases. Besides, the treatment of mastitis has become more difficult due to the emergence of antibiotic-resistant strains of bacteria. Therefore, new biomarkers for this disease urgently needed to identify that are effective in early diagnosis and possible drug targets to help design novel therapeutic interventions within veterinary and animal science areas. The use of bioinformatics and machine learning methods is significant in finding potential biomarkers for mastitis decease. Advanced technologies like microarray and RNA sequencing (RNA-seq) allow for rapid and comprehensive analysis of gene activity across the entire genome. Technology have made a lot of progress in recent times opening new avenues for critical care medicine at precision levels to more effectively understand how different biological processes function on the molecular scale [4,5]. There have been several research identified potential biomarkers for mastitis disease [6–8].

**Fig 1:**
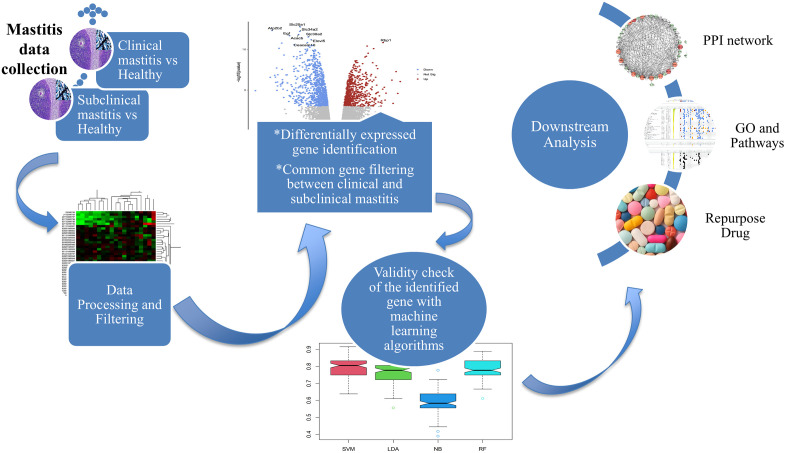
A schematic workflow of this study. This study began with preprocessing microarray and RNA-seq count data for both clinical and subclinical mastitis and analyzed DEGs with machine learning validation and performed downstream analyses.

There have been several research is going on finding the biomarkers and drug targets [9–12]and so the study progressing to discover the genes linked to mastitis in dairy cattle have combined the use of microarray meta-analysis [13] and RNA-seq data [14] together with some machine-learning algorithms. Several studies in the literature have focused on discovering biomarker genes for either clinical nor subclinical mastitis [15,16], but no one shown the clinical and subclinical interaction with drug targets identification and machine learning validation. In this study, we identified the significant biomarkers and pathways between the clinical and subclinical mastitis disease as well as potential drug targets and repurpose existing drugs detected for the early treatment of subclinical mastitis together with clinical mastitis disease with machine-learning (ML) validation approaches. A schematic workflow for analyzing differentially expressed genes (DEGs) using clinical and subclinical mastitis data in this study presented in Fig 1. The identified biomarkers and drug targets could greatly improve mastitis disease management and deepen our understanding of the condition.

## 2. Methods and materials

### 2.1. Data processing and differential expression analysis using combinatorial statistical methods

The RNA-Sequencing and microarray-based gene expression transcriptomics datasets for mastitis disease for both clinical and subclinical were collected from the NCBI Gene Expression Omnibus (GEO). Needed to preprocess these datasets before doing necessary analysis, excluding lowly expressed genes, impute missing values and log2 transformation with quantile normalization. and to remove the technical variability across studies, batch effects reduction was applied to facilitate robust gene expression analysis [14]. We used *removeBatchEffect* function to adjust the batch effects of each dataset [14]. Datasets were analyzed separately for identification of differentially expressed genes (DEGs). The *DESeq2* approach [17] for RNA count data and *limma* [18] for microarray datasets have been applied to identify the DEGs. We considered only those common DEGs in all datasets with satisfying the statistical threshold parameters, adjusted p-value <0.05 along with $|{log}_{\text{2}}\text{ fold change }|\geq \text{1}\;$ [19,20].

### 2.2. Validation of the selected genes with machine learning algorithms

The identified genes have been validated with four popular supervised machine leaning (ML) classification algorithms, support vector machine (SVM) [21], naïve bayes (NB) [22] linear discriminant analysis (LDA) [23] and random forest (RF) [24]. We validated the identified biomarkers in two ways, firstly used internal validation process and for that we randomly select one dataset for train set and another dataset for test set each of with only commonly identified genes, secondly an external dataset used as test data with commonly identified gene. The accuracy, sensitivity, specificity, positive predictive value (PPV), negative predictive value (NPV) and detection rate (DR) will be analyzed to check the validity of the selected genes [19].

### 2.3. Protein-protein interaction network analysis

STRING- a widespread web-based tool utilized for analyzing Protein-protein interaction (PPI) [25] and the network have been visualized with Cytoscape [26]. A PPI network is provided by STRING which represents the functional and physical relationships among some of the differentially expressed genes (DEGs) when their protein counterparts are used as input. In this analysis, a PPI network have been constructed using information from STRING, and further visualized and analyzed through the Cytoscape open-source platform. The hub proteins of the PPI network determined with the help of maximum degree connectivity scores in this analysis via the CytoHubba plugin [27] in Cytoscape. The higher the values of degree connectivity for nodes, more edges are attached in those hub proteins [19].

### 2.4. Gene ontology and pathway enrichment analysis

We explored the roles and signaling pathways of commonly differentially expressed genes by conducting Gene Ontology (GO) as well as pathway enrichment studies with *g:Profiler*, a well-known gene set enrichment web tool [28]. The GO terms cover three main categories namely, biological processes (BP), molecular functions (MF) and cellular components (CC). For pathway analysis, this study relied on the KEGG (Kyoto Encyclopedia of Genes and Genomes) databases [29]. We considered GO terms and pathways as significantly enriched when their adjusted p-value was below 0.05 [19,30].

### 2.5. Drug-gene interaction analysis

The drug-gene interaction analysis comprised of a widespread DSigDB (version 1.0) database for candidate drug identification which is highly related to selected DEGs [31]. The mastitis biomarker genes and pathways we found in cows are well-characterized human orthologs, so we tapped DSigDB to pick out a handful of promising repurposed drugs. Even though DSigDB is built for human genes, we mapped cattle gene equivalents to find drugs that could be repurposed for immune pathways, bridging the gap until cattle-specific resources expand. The DSigDB is a freely accessible web-based resource with detailed drug and comprehensive drug target information. DSigDB gene sets provide seamless integration with GSEA software, enabling researchers to connect gene expressions with various drugs and compounds. The database includes 22,527 gene sets, covering 17,389 unique drugs linking to 19,531 genes, making it a valuable resource for drug regeneration and translational research. The network of DEGs and the candidate drugs identified by the DSigDB database is implemented via Enrichr [32].

## 3. Results

### 3.1. Differential expression analysis

**Fig 2:**
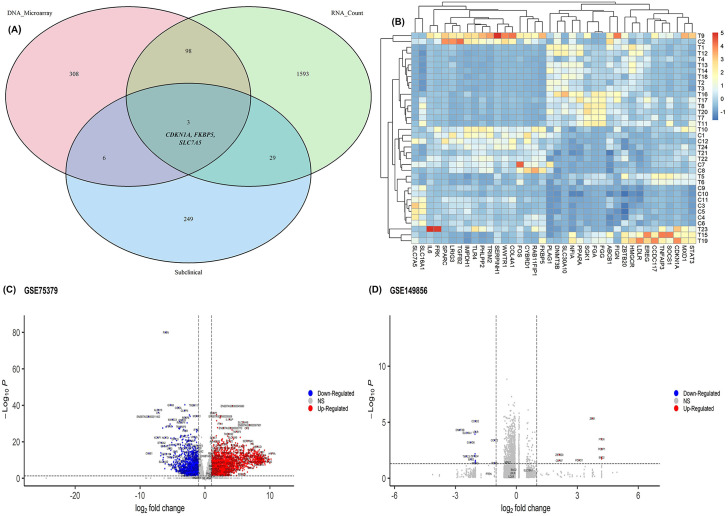
Identification of common DEGs across clinical and subclinical mastitis datasets. A) 38 common genes identified among clinical (Microarray and RNA count) and Subclinical mastitis datasets. Three genes (CDKN1A, FKBP5 and SLC7A5) have been identified as common among all the datasets; B) heatmap plot for the common genes in GSE75379 dataset. The graph indicated the presence of differentially expression in diseases groups than control groups; C) volcano plots for the GSE75379 and D) GSE149856 datasets with indicating the presence of upregulated (red color) and downregulated (blue color) genes. The gray color indicated the equally expressed genes.

The RNA-Sequencing transcriptomics and microarray gene expression datasets for both clinical and subclinical mastitis disease have been collected from the NCBI Gene Expression Omnibus (GEO) [33]. Table 1 described the data used in this study. After collecting datasets from GEO, we keep only those genes whose sum of all CPM value more than 2 for the RNA-seq count datasets. The differentially expressed genes have been identified with *limma* for microarray datasets and *DESeq2* for RNA-seq count datasets. The volcano plots for each dataset clearly indicated the presence of upregulated (red color) and downregulated (blue color) genes (Figs 2C, S1).

**Table 1:** Data description used in this study.

Accession no.	Species	Bacteria	Source	Types	Platform	Disease Type	Samples^*^ (C:T)	References
GSE15025	*B. taurus*	Escherichia coli	mammary gland	Microarray	Affymetrix	Clinical Mastitis	15:15	[34]
GSE24217	*B. taurus*	*E. coli*	mammary gland	Microarray	Affymetrix	Clinical Mastitis	23:26	[35]
GSE24560	*B. taurus*	*E. coli*	pbMEC^a^	Microarray	Affymetrix	Clinical Mastitis	27:31	[36]
GSE50685	*B. taurus*	*E. coli*	mammary gland	Microarray	Affymetrix	Clinical Mastitis	5:15	[37]
GSE75379	*B. taurus*	*E. coli*	mammary gland	RNA Read Count	Illumina	Clinical Mastitis	6:12	[38]
GSE159286	*B. taurus*	*E. coli*	Blood	RNA Read Count	Illumina	Clinical Mastitis	53:52	[39]
GSE149856	*B. taurus*	*E. coli*	Milk	RNA Read Count	Illumina	Subclinical Mastitis	9:18	[40]

a pbMEC: Primary bovine mammary gland epithelial cells.

* Number of healthy (C) and infected samples (T).

### 3.2. Detection of shared differentially expressed genes

We selected our target genes those are common between microarray clinical mastitis datasets and RNA-seq count subclinical mastitis data or RNA-seq count clinical mastitis datasets and RNA-seq count subclinical mastitis data (Fig 2A). We finally selected 38 genes between clinical and subclinical mastitis datasets. These genes are used for further analyses. Three genes (***CDKN1A***, ***FKBP5*** and ***SLC7A5***) are identified as common among all clinical and subclinical mastitis datasets. The heatmap plot using *pheatmap* r package in Fig 2B indicated the differentially expression patters of the 38 genes over different sample. These genes are highly expressed in treatment groups then control groups.

### 3.3. Validation of the selected genes with machine learning algorithms

**Fig 3:**
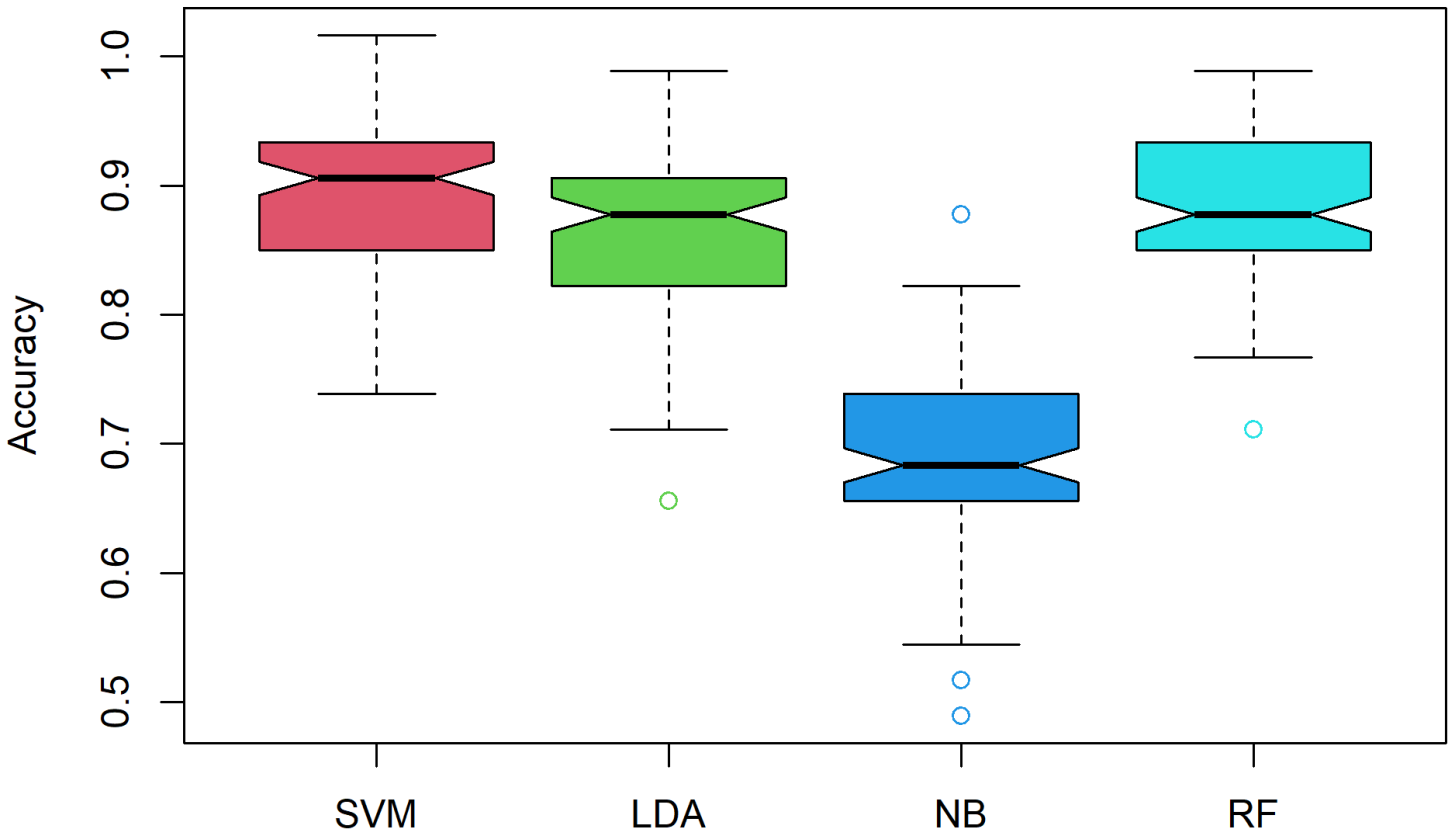
Accuracy boxplot for four machine learning classifiers on internal validation. We used randomly selected dataset, GSE159286 as training set and GSE149856 as test set. We used 10-fold cross validation across four classifiers (SVM, LDA, NB and RF) and find almost 90% accuracy for all classifiers that indicated the validity of our commonly identified genes among clinical and subclinical mastitis datasets.

The validation of the identified 38 genes, we applied four classification algorithms, support vector machine (SVM), linear discriminant analysis (LDA), naïve bayes (NB) and random forest (RF). For internal validation, we used one RNA-seq count dataset of clinical and one for subclinical mastitis for this analysis (randomly select GSE159286 as training sets and GSE149856 as test set). The identified available common genes have been used in both training and test data for machine learning classifiers. The mastitis or control groups have been used in both dataset as the classes for classification. The results of this ML analyses with different matrices shown in Table 2 and in Fig 3 concluded that, the identification of the common genes among different datasets are valid, since almost 90% accuracy carried for all classification algorithms. For external validation, GSE159286 was used as the training dataset and the external GSE51856 as the independent test dataset. GSE51856 is an RNA seq study of bovine milk and blood monocytes that examines the host response to Streptococcus uberis, a major cause of mastitis in dairy cows. In this work, only the milk samples from GSE51856 were analyzed, comprising 44 samples in total, with 25 healthy controls and 19 infected cases. The results of the machine learning analyses using different datasets, as presented in S1 Table and S2 Fig, confirm the validity of identifying common genes across datasets. This is evidenced by the consistently high accuracy of nearly 90% across all classification algorithms. The accuracy, sensitivity, specificity, negative predictive value (NPV), positive predictive value (PPV) and detection rate (DR)results validate the identified genes among the datasets.

**Table 2:** Different matrices for ML classification algorithms based on internal test data.

	Accuracy	Sensitivity	Specificity	PPV	NPV	DR
**SVM**	0.896	0.672	0.905	0.859	0.764	0.672
**LDA**	0.869	0.700	0.831	0.779	0.765	0.700
**NB**	0.685	0.943	0.281	0.528	0.861	0.943
**RF**	0.889	0.718	0.853	0.808	0.780	0.718

### 3.4. PPI network analysis with hub genes detection

**Fig 4:**
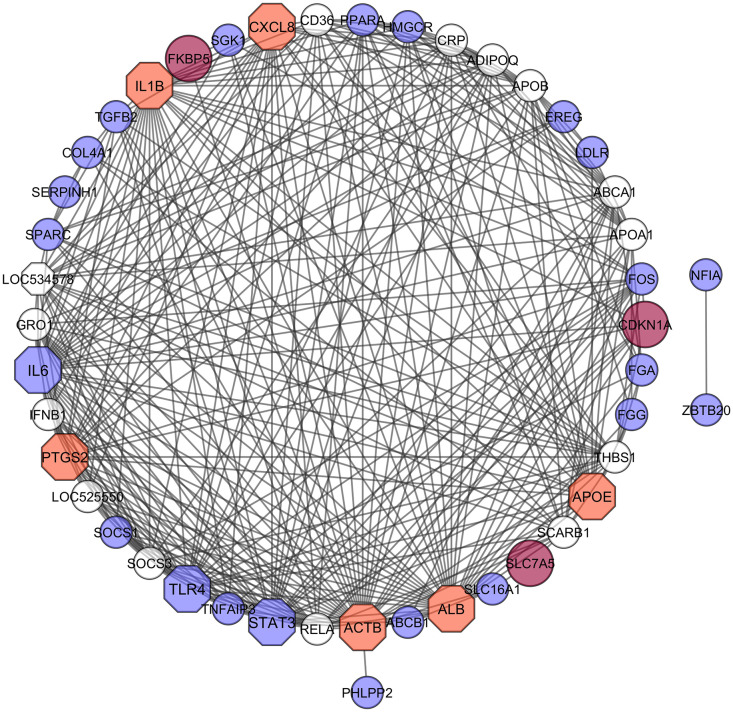
The protein-protein interaction analysis for the common genes identified among clinical and subclinical mastitis datasets. The PPI network analysis indicated the interconnection among the identified common genes. The larger the sizes of the octagons indicated as highly connected genes. Ten hub proteins (ACTB, ALB, IL6, IL1B, STAT3, TLR4, PTGS2, APOE, CXCL8, LOC534578, CDKN1A, FKBP5, and SLC7A5) were identified based on the highest degree connectivity.

To identify the signaling biomolecules, we analyzed PPI network around the proteins encoded by common 38 DEGs (Fig 4). The topological analysis identified 10 hub proteins (i.e., ACTB, ALB, IL6, IL1B, STAT3, TLR4, PTGS2, APOE, CXCL8, LOC534578/ VCAM1, CDKN1A, FKBP5 and SLC7A5) based on the top degree connectivity. In Fig 4, the larger the size of the nodes indicates highly connective genes, and the octagons indicates those genes that’s are present both in clinical and subclinical mastitis. The overview of these 10 hub genes briefly described in Table 3.

**Table 3:** Summary description of the 10 hub genes.

Gene Name	description	Regulation
ACTB	actin beta	Down
ALB	albumin	Up
IL6	interleukin 6	Up
IL1B	interleukin 1 beta	Down
STAT3	signal transducer and activator of transcription 3	Up
TLR4	toll like receptor 4	Down
PTGS2	prostaglandin-endoperoxide synthase 2	Down
APOE	apolipoprotein E	Up
CXCL8	C-X-C motif chemokine ligand 8	Down
LOC534578/ VCAM1	vascular cell adhesion molecule 1	Down
CDKN1A	cyclin dependent kinase inhibitor 1A	Up
FKBP5	FKBP prolyl isomerase 5	Up
SLC7A5	solute carrier family 7 member 5	Up

### 3.5. Functional annotation analysis

**Fig 5:**
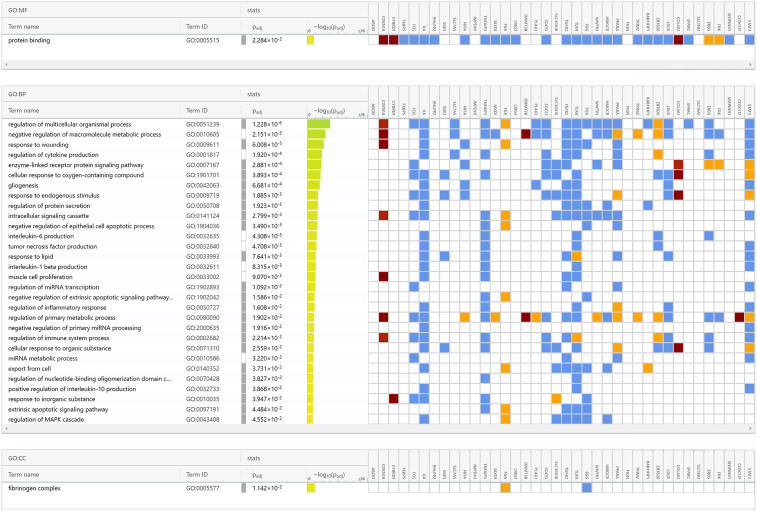
The GO enrichment analysis for the commonly identified genes. This figure shown the important enriched terms for each Biological Process (BP), Molecular Function (MF) and Cellular Component (CC) for the 38 commonly identified genes among clinical and subclinical mastitis datasets. The color intensity indicated the enrichment significance of the connected genes across the GO terms.

We performed gene set enrichment analysis to get a clearer picture about the common differentially expressed genes (DEGs) in mastitis. Our findings revealed these genes play a significant role in biological processes, including the regulation of multicellular organismal processes, “cell population proliferation”, “response to wounding”, “intracellular signaling pathways”, and the “regulation of epithelial cell apoptosis”. In terms of molecular function, the DEGs were notably associated with the “protein binding” activity. Additionally, these genes were significantly enriched in the cellular component known as the “fibrinogen complex”. A summary of the top Gene Ontology terms is presented in Fig 5.

**Fig 6:**
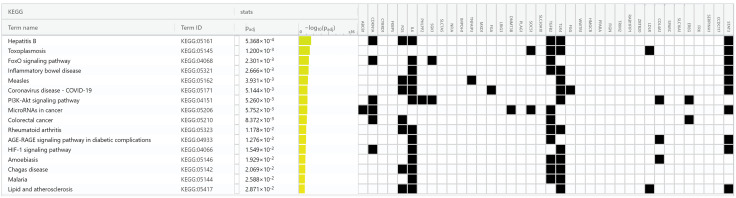
The KEGG pathways analysis for the 38 common genes. This figure shown the significant pathways for the commonly identified genes among clinical and subclinical mastitis datasets. The color intensity indicated the significance of the connected genes across the pathways.

We performed a pathway analysis to pinpoint the pathways that were dysregulated and enriched with common DEGs. Our analysis identified several pathways including “Hepatitis B”, “Toxoplasmosis”, “FoxO signaling pathway”, “Inflammatory bowel disease”, “Measles”, “Malaria” and “HIF-1 signaling pathway”. The top terms KEGG pathways are summarized in Fig 6.

### 3.6. Drug prediction analysis

The statistically significant repurpose drugs have been identified via DSigDB database for the common DEGs between clinical and subclinical mastitis. In Table 4, we have summarized the top 10 significant drugs based on adjusted p-values, odd ratio and combined score that had interaction with common DEGs. The dexamethasone, cycloheximide, ACMC-20mvek, 5-Fluorouracil, simvastatin, aspirin, N-Acetyl-L-cysteine, tetradioxin are the significantly identified drugs in this study to treat subclinical and clinical mastitis disease.

**Table 4:** Summary description of the top 10 repurposing drugs.

Term	P-value	Adjusted P-value	Odds Ratio	Combined Score
dexamethasone	1.07E-11	1.54E-08	22.0647	557.3884609
cycloheximide	1.39E-11	1.54E-08	25.57555	639.4160879
ACMC-20mvek	7.02E-10	5.19E-07	48.24246	1016.838444
5-Fluorouracil	1.84E-09	1.02E-06	10.31556	207.4603716
simvastatin	4.26E-09	1.72E-06	20.69001	398.7780623
aspirin	4.64E-09	1.72E-06	14.37926	275.9038655
N-Acetyl-L-cysteine	5.81E-09	1.84E-06	19.93509	378.0556984
resveratrol	1.08E-08	2.71E-06	8.432234	154.7146873
CHROMIUM	1.10E-08	2.71E-06	31.74262	581.7779007
Tetradioxin	1.61E-08	3.57E-06	6.639804	119.1578076

## 4. Discussion

Clinical as well as subclinical mastitis in dairy cattle is a severe problem with wide-ranging social, cultural, economic, and human repercussions in addition to the animals. By successfully identifying potential new biomarkers for clinical and subclinical mastitis, it is possible to revolutionize mastitis diagnostics and management in dairy farming. Early detection of mastitis and the ability to differentiate between clinical and subclinical forms can help farmers implement targeted intervention strategies, reducing the economic losses associated with this disease. In this study, ML and system biology approaches implemented to identify common genes, their associate GO, pathways and therapeutic drug target for leveraging both clinical and subclinical mastitis disease.

Compared to earlier bioinformatics reports in mastitis research, which relied on the identification of genes by differential analysis, our approach is very different. Nevertheless, we have implemented methodologies, particularly gene expression analysis, which provides key gene [19]. Most of the previous reports detected the biomarker genes either from clinical mastitis or subclinical mastitis [8,14,41–43]. We used our proposed pipelines in both clinical and subclinical forms of mastitis datasets and tried to find out the biomarkers associated with both of the types. Some of the previous reports use only p-value to detect biomarkers. If we only utilize one method, such as the p-value, there is a chance that we will find some unsuitable genes as the biomarker [19]. And the p-value needs to adjust with some adjustment methods to control the increased risk of false positives (Type-I errors). We applied Benjamini-Hochberg adjusted p-value along with fold change values to detect biomarkers from both clinical and subclinical mastitis in dairy cattle. Unlike prior studies, we validated via machine learning classifiers. The performance of state-of-the-art methods was evaluated the classification performances in which obtained higher score in accuracy, specificity and sensitivity.

Among the identified 13 potential biomarkers for both clinical and subclinical mastitis diseases, the Cyclin Dependent Kinase Inhibitor 1A or p21 (CDKN1A) gene plays a significant role in the cell cycle and responding to DNA damage. Its expression varies in the mammary epithelium of lactating Holstein cows that produce exceptionally high or low milk protein and fat percentages [44]. FKBP5 (FK506 Binding Protein 5) involved in the regulation of the glucocorticoid receptor and stress response. Recent gene expression comparisons between uninfected quarters of healthy cows and those with mastitis also identified FKBP5 as a differentially expressed gene [45,34]. It may influence the inflammatory response and immune function during mastitis. SLC7A5 (Solute Carrier Family 7 Member 5) is an amino acid transporter involved in cellular uptake of essential amino acids. It may be a factor in cell proliferation and immune responses during mastitis [46]. The SLC7A5 transporter gene use for granule cell development [47] and that may play a significant role in mastitis tissue development.

Interleukin 6 (IL-6) is a cytokine that contributes to both inflammation and B cells maturation. Its expression is elevated in mastitis, playing a role in the inflammatory response and maintaining immune homeostasis. Monitoring IL-6 levels in milk can aid in the early detection of mastitis, which is particularly crucial in cases of subclinical inflammation [48]. ACTB, also known as beta-actin, is a gene common to human organism that is mostly expressed and thus can serve as a reference in some gene expression studies. The ALB gene encodes serum albumin which is in high concentration in human blood [49]. Serum albumin regulates osmotic pressure and transports small molecules such as fatty acids at the same time. It also has an involvement in mastitis inflammatory process.

For immune response towards infections IL1B is a pro-inflammatory cytokine which plays an important role. The inflammatory development of mastitis relies on this [42]. IL6 can activate transcription factor called STAT3 (Signal Transducer and Activator of Transcription 3). In mastitis it contributes to its development and reduces milk production by dairy cows [50]. Bacterial lipopolysaccharides are known by TLR4 (Toll-Like Receptor 4), which activates innate immune responses. This gene is a great candidate marker for mastitis resistance because it plays a role in neutrophil migration into and out of the mammary gland throughout the infection [51].

PTGS2 (Prostaglandin-Endoperoxide Synthase 2; COX-2) is an enzyme that transforms arachidonic acid into prostaglandins which are accountable in inflammatory reaction. This gene is downregulated during inflammation of mastitis [52]. Apolipoprotein E (APOE) is a protein engaged in lipids transport as well as immune reaction. This also hinted a possible role during immune response to mastitis and alteration of inflammation. CXCL8 (C-X-C motif chemokine ligand 8; IL-8) serves as the chemokine responsible for recruiting neutrophils. It has been shown previously that this gene stood out among the three highest ranked genes supposed to be associated with E. coli mastitis [13]. VCAM1 takes part in the gathering of leukocytes around inflammation sites. In addition, it has been detected in lymph nodes from subclinical mastitis dairy cows [53]. In this manner, these genes intervene in different dimensions of resistance against disease, inflammatory responses and cellular activities leading to occurrence as well as development of bovine mastitis.

The identified GO and KEGG pathway enrichments terms could lead to the emergence of novel therapeutic approaches and vaccines to combat mastitis effectively. The identified “Response to wounding” Go term emerges as the most critical factor in mastitis progression in cows, directly driving the acute inflammatory reaction to bacterial invasion [54]. By promoting the quick growth of immune cells like neutrophils and macrophages, which support the inflammatory response and tissue healing after infection, “cell population proliferation” plays a crucial part in the development of mastitis in cows [7]. The “Intracellular signaling pathways” in mastitis progression propagate critical signals that coordinate immune cell activation, cytokine production, and pathogen defense within mammary cells [55,14]. The FoxO and HIF-1 signaling pathways directly influence in mastitis through infected mammary tissues by regulating inflammation, oxidative stress, immune responses and cell survival [56,57].

The identified mastitis biomarker genes and pathways we found in cows are well-characterized human orthologs, so we used DSigDB to identify promising repurposed drugs. DSigDB lacks native bovine gene mappings, requiring manual ortholog conversion that may introduce inaccuracies in veterinary drug repurposing predictions. This study lacks experimental validation of the biomarkers or therapeutic claims including the data source variation with small size sample collected different time. We suggest carrying out more biological and clinical research to explore whether the candidate drugs could be repurposed to treat mastitis. While bioinformatics and systems biology have been invaluable in this research, it's important to validate these findings clinically before they can be used as reliable biomarkers. Large-scale transcriptomic studies covering clinical and subclinical mastitis cases in dairy cows will be carried out in the future through clinical partnerships with veterinary facilities.

## 5. Conclusions

This study demonstrated the power of bioinformatics in advancing our understanding and management of mastitis. Through comprehensive analysis of transcriptomic data, this study identified several candidate new biomarkers and potential drug targets that could revolutionize the diagnosis and treatment of both clinical and subclinical forms of this disease. Furthermore, this study highlighted how combining machine learning and bioinformatics helps pinpoint the most promising genes that might be key candidate biomarkers for both clinical and subclinical mastitis. The identified key biomarkers could be transformed into diagnostic methods, which would allow for early and accurate identification of mastitis in both clinical and subclinical form. Thus enhancing prognosis and lessening the financial load on dairy farming by early detection and controlling with treatments of mastitis in subclinical stage. In order to apply these discoveries in the real world further experimental validation and clinical trials remain important. Additionally, our study helps to improve health care for dairy cattle by supports developing strategies for early detection and effective treatment against udder infections thereby saving the farmers from heavy financial losses as well as boosting their milk production.

## Supporting information

S1 Fig Volcano plots for the A) GSE15025, B) GSE24217, C) GSE24560, D) GSE50685 and E) GSE159286 datasets. This figure indicating the presence of upregulated (red color) and downregulated (blue color) genes in each dataset. The gray color indicated the equally expressed genes.
(TIFF)

S2 Fig Accuracy boxplot for four machine learning classifiers on external validation. (TIF)

S1 Table Different matrices for ML classification algorithms based on External test data. (DOCX)
