# Supplementary figures and images for: Bioinformatics approach to identify potential biomarker and drug target for the clinical and subclinical mastitis disease in dairy cattle
Source: PLoS One. 2026 May 13;21(5):e0349172. doi: 10.1371/journal.pone.0349172 (PMC13170882; doi:10.1371/journal.pone.0349172)

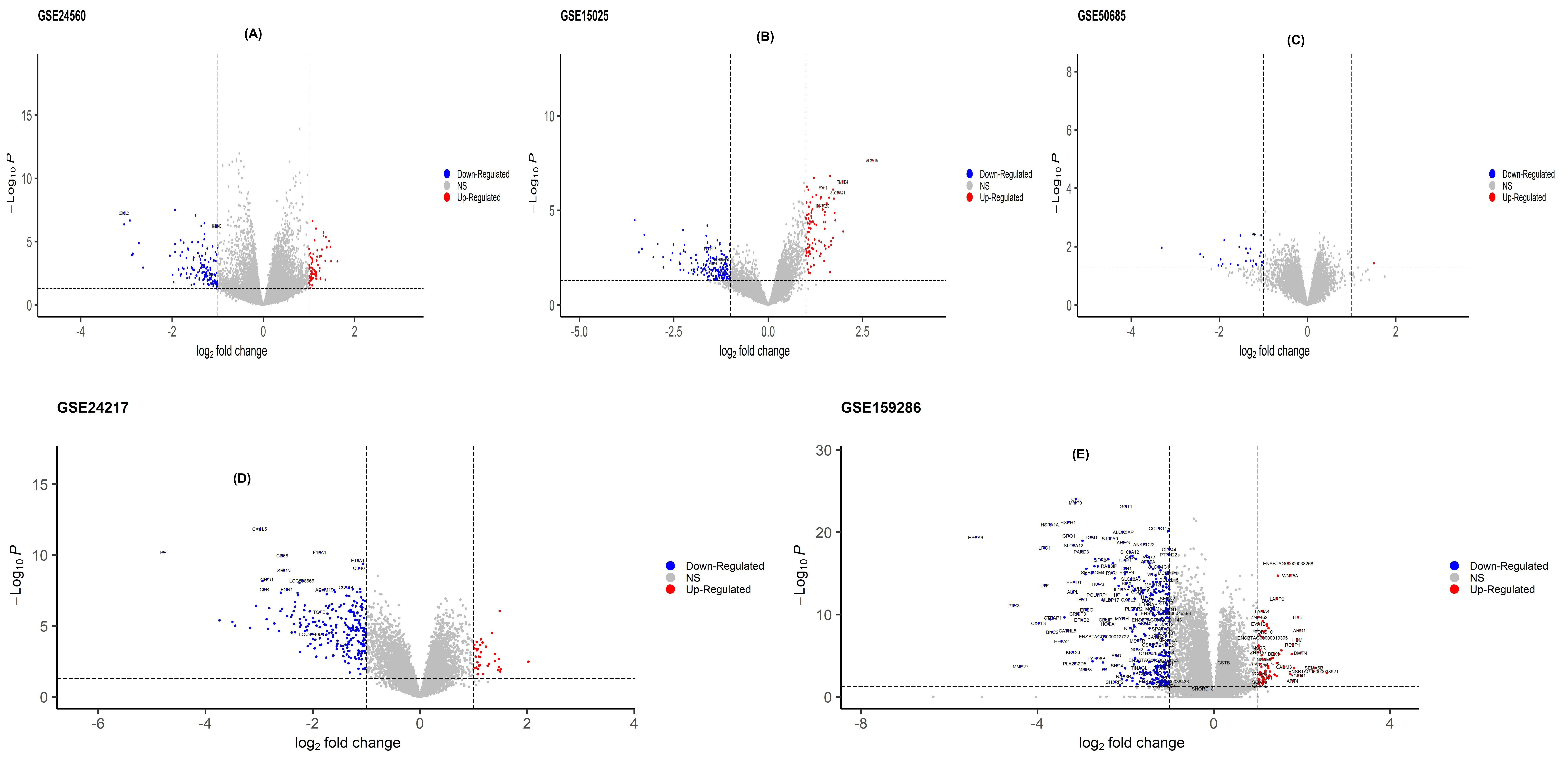

Supplement: S1 Fig — This figure indicating the presence of upregulated (red color) and downregulated (blue color) genes in each dataset. The gray color indicated the equally expressed genes. (TIFF) [file pone.0349172.s001.tiff]

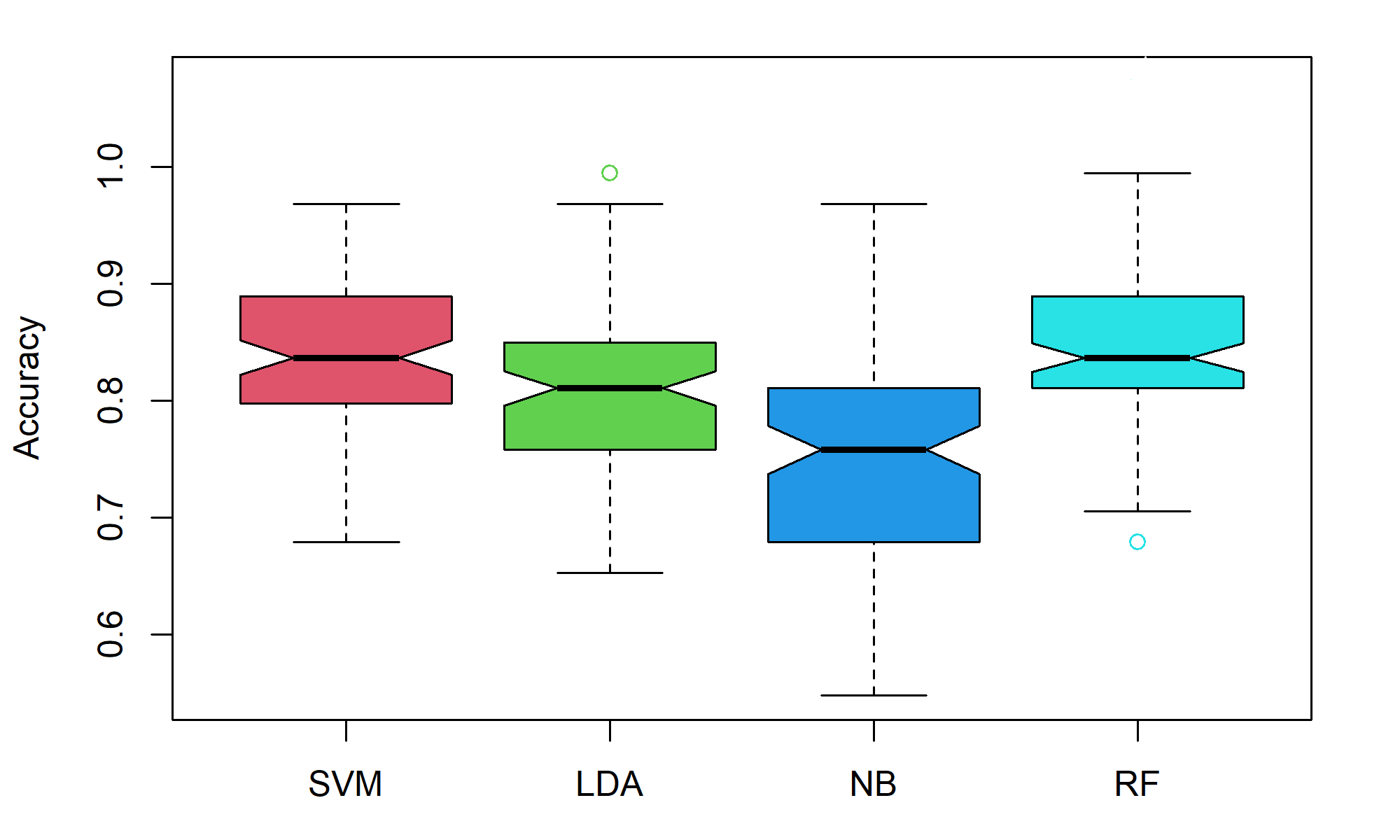

Supplement: S2 Fig — (TIF) [file pone.0349172.s002.tif]
