# Supplementary material for: Bioinformatics approach to identify potential biomarker and drug target for the clinical and subclinical mastitis disease in dairy cattle
Source: PLoS One. 2026 May 13;21(5):e0349172. doi: 10.1371/journal.pone.0349172 (PMC13170882; doi:10.1371/journal.pone.0349172)
Supplement: S1 Table — (DOCX) [file pone.0349172.s003.docx]

**Table S1. Different matrices for ML classification algorithms based on External test data**

|  | **Accuracy** | **Sensitivity** | **Specificity** | **PPV** | **NPV** | **DR** |
| --- | --- | --- | --- | --- | --- | --- |
| **SVM** | 0.836 | 0.798 | 0.880 | 0.872 | 0.809 | 0.798 |
| **LDA** | 0.805 | 0.806 | 0.809 | 0.821 | 0.798 | 0.806 |
| **NB** | 0.746 | 0.628 | 0.874 | 0.819 | 0.708 | 0.628 |
| **RF** | 0.838 | 0.848 | 0.834 | 0.849 | 0.835 | 0.848 |
